# Estimation of the Vertical Distribution of Radiocesium in Soil on the Basis of the Characteristics of Gamma-Ray Spectra Obtained via Aerial Radiation Monitoring Using an Unmanned Helicopter

**DOI:** 10.3390/ijerph14080926

**Published:** 2017-08-17

**Authors:** Kotaro Ochi, Miyuki Sasaki, Mutsushi Ishida, Shoichiro Hamamoto, Taku Nishimura, Yukihisa Sanada

**Affiliations:** 1 Fukushima Environmental Safety Center, Japan Atomic Energy Agency, 45-169 Sukakeba, Kaibama-aza, Haramachi, Minamisoma, Fukushima 975-0036, Japan; sasaki.miyuki@jaea.go.jp (M.S.); sanada.yukihisa@jaea.go.jp (Y.S.); 2 NESI, Inc., 1-9-3, Saku-machi, Taira-aza, Iwaki, Fukushima 970-8026, Japan; ishida.mutsushi@jaea.go.jp; 3 Graduate School of Agricultural and Life Sciences, The University of Tokyo, 1-1-1 Yayoi, Bunkyo-ku, Tokyo 113-8657, Japan; shoichi@soil.en.a.u-tokyo.ac.jp (S.H.); takun@soil.en.a.u-tokyo.ac.jp (T.N.)

**Keywords:** Fukushima Daiichi Nuclear Power Plant accident, aerial radiation monitoring, unmanned helicopter, radiocesium, vertical distribution

## Abstract

After the Fukushima Daiichi Nuclear Power Plant accident, the vertical distribution of radiocesium in soil has been investigated to better understand the behavior of radiocesium in the environment. The typical method used for measuring the vertical distribution of radiocesium is troublesome because it requires collection and measurement of the activity of soil samples. In this study, we established a method of estimating the vertical distribution of radiocesium by focusing on the characteristics of gamma-ray spectra obtained via aerial radiation monitoring using an unmanned helicopter. The estimates are based on actual measurement data collected at an extended farm. In this method, the change in the ratio of direct gamma rays to scattered gamma rays at various depths in the soil was utilized to quantify the vertical distribution of radiocesium. The results show a positive correlation between the abovementioned and the actual vertical distributions of radiocesium measured in the soil samples. A vertical distribution map was created on the basis of this ratio using a simple equation derived from the abovementioned correlation. This technique can provide a novel approach for effective selection of high-priority areas that require decontamination.

## 1. Introduction

In 2011, the Fukushima Daiichi Nuclear Power Plant (FDNPP) accident occurred following a catastrophic earthquake and subsequent tsunami. Many institutes and universities have investigated the deposition density of radionuclides and dose rate above the ground via ground measurements (e.g., [1]). The results of these measurements have elucidated the contamination situation for radionuclides with a relatively long half-life, such as ^134^Cs (T_1/2_: 2 years) and ^137^Cs (T_1/2_: 30 years), in the soil around FDNPP. Although more than six years have passed since the accident occurred, it is still necessary to evaluate the physicochemical properties of radiocesium in the soil (^134^Cs and ^137^Cs) in order to monitor the long-term behavior of radiocesium in the environment.

Shortly after the accident, the U.S. Department of Energy and the Japan Ministry of Education, Culture, Sports Science and Technology immediately performed aerial radiation monitoring (ARM) using a manned helicopter to evaluate the distribution of Fukushima-derived radionuclides in the Fukushima Prefecture [2,3]. The temporal variation of the dose rate distribution around FDNPP has been continuously monitored by the Nuclear Regulation Authority in Japan and the Japan Atomic Energy Agency (JAEA) to evaluate the short half-life decay of radionuclides resulting from the decontamination efforts [4]. The analytical schema of ARM using a manned helicopter has been improved in response to geographical and weather conditions [5,6]. ARM using an unmanned helicopter is suitable for small-scale monitoring and evaluation of the local distribution of radionuclides and dose rate above the ground within a narrow area because as per Japanese Aviation Law, a manned helicopter cannot fly at altitudes lower than 150 m. ARM using an unmanned helicopter allows for low-altitude evaluation of the detailed deposition of radionuclides on surface soil. We measured the local dose rate [7,8] and the ^134^Cs/^137^Cs ratio [9] within an 80 km^2^ area around FDNPP using an unmanned helicopter.

Estimating the vertical distribution of radiocesium in soil can provide important information for understanding the migration mechanism of radiocesium in the surface soil over time. The measurement results obtained by several institutes immediately after the accident for radiocesium deposition on the soil around FDNPP indicate that most of the radiocesium migrated within the surface soil [10,11,12,13,14]. The penetration depth of radiocesium in the soil was influenced by soil characteristics such as particle size, clay content, and organic carbon content. It was found that ^137^Cs penetrated to a greater depth in forest soils compared with other types of soil [14]. Sato et al. (2015) reported the migration of radiocesium toward deeper layers in sandy soils affected by the tsunami [15]. These measurement results indicate that the initial vertical distribution of radiocesium in the soil changed as a function of soil characteristics and the surrounding environment. The vertical distribution of radiocesium in gray lowland soils, croplands, grasslands, and forest soils before and after the first rainy season was unchanged because the majority of radiocesium was not lost from the soil in a insoluble form [16,17]. In some cases, long-term investigations of the temporal variation over a wide area showed a downward migration of radiocesium to deeper sites over time [18,19]. Thus, continuous monitoring of the vertical distribution of radiocesium in soil is needed not only for proposing effective decontamination methods but also increasing the knowledge in terms of geoscience and radiochemistry. Previous studies on the vertical distribution of radiocesium in soils have focused on accurately evaluating the differences in the distribution under various types of land uses as well as the temporal variation in a selected area. The typical method used for measuring the vertical distribution of radiocesium is troublesome because it requires collection and measurement of the activity of soil samples. Thus, a simpler and more rapid estimation method capable of effectively identifying high-priority radiocesium decontamination areas around FDNPP, known as “evacuation order zones”, is required. 

In this study, we attempt to develop a method for evaluating the vertical distribution of radiocesium using ARM with an unmanned helicopter. This method is based on the relationship between the ratio of direct gamma rays to scattered gamma rays and radiocesium depth in the soil. We applied the proposed method in an extended farm to verify our assumption that the gamma-ray spectra obtained via ARM are representative of the vertical distributions of radiocesium in soil. Soil samples were collected and evaluated to confirm the validity of the ARM results. Our method could provide a novel approach for effective selection of high-priority areas that require decontamination.

## 2. Materials and Methods

### 2.1. Study Site and Data Correction Conditions

**Figure 1:**
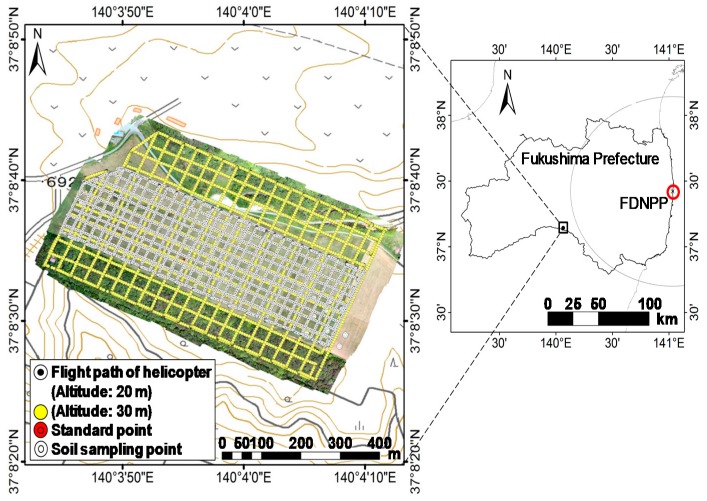
Location of the study site and measurement points. Yellow circles indicate the survey lines of ARM, and white circles indicate the soil sampling points.

The study site is located in the central part of the Nishigo village in the Fukushima Prefecture, which is located approximately 100 km southwest of FDNPP. The site is 600 m in length and 180 m in width. The farm belongs to the National Livestock Breeding Center in Japan. A photo of the study site is shown in Figure 1. The maps created in this paper are based on the GSI map published by the Geospatial Information Authority of Japan. With the exception of the southern area, the entire decontamination work, known as “inversion tillage”, was performed in September 2012 using a plow. Currently, radiocesium exists mainly in the deeper soil because the inversion tillage performed with the purpose of decontamination mixed the surface soil with deeper soil. 

**Figure 2:**
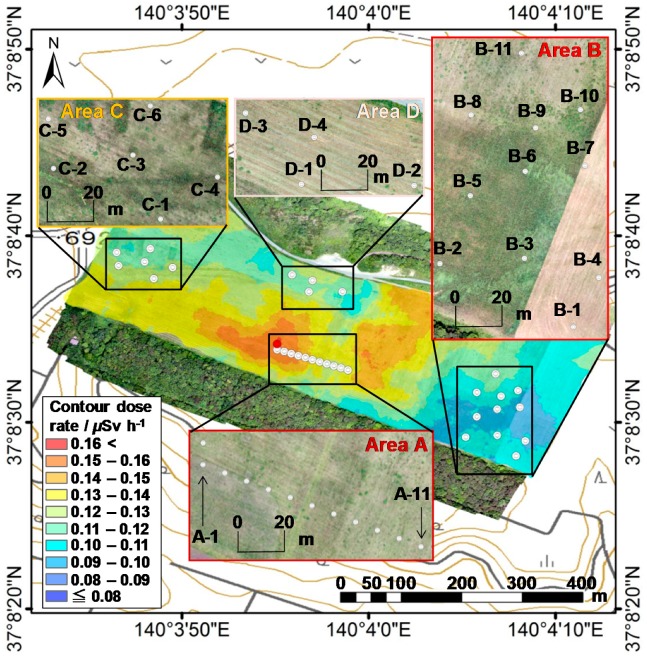
Contour map of the dose rates was derived from the measurement results using a handheld survey meter on the ground.

Dose rates were measured at altitude of 10 cm above the ground level (agl.) using a handheld survey meter that use a CsI detector (NESI Co., Ltd., Ibaraki, Japan). Contour map of the dose rates is shown in Figure 2. The map was created via Kriging method that is one of the method of interpolating the value. The dose rates were interpolated based on the value on the surrounding 36 neighboring points. The distance from the center point to each neighboring point were not settled. The range of values was between 0.080 and 0.16 μSv h^−1^. The dose rate in Area A was relatively high in the farm because inversion tillage was not performed in this area. In contrast, the dose rates in Areas B, C, and D were relatively low in the farm where inversion tillage was performed.

ARM using an unmanned helicopter was performed on 10 June 2016. An unmanned helicopter, R-MAX G1 (Yamaha Co., Ltd., Shizuoka, Japan), was developed for spraying pesticides on paddy fields. A Differential Global Positioning System (GPS) was used to obtain the position data of the helicopter. Deposition of radiocesium on the ground was measured in the terrain below the flight path; this measurement was performed at 20 and 30 m agl. The helicopter flight path is shown in Figure 1. Its flight speed varied between 4.0 and 5.0 m s^−1^. The flight intervals at 20 and 30 m agl. were 20 and 30 m, respectively. 

Soil sampling was performed on 4 and 5 July 2016, to confirm the validity of the ARM results. Four ground sampling areas were selected from the study site using advanced ground measurement results. The soil sampling points are shown in Figure 2. We obtained 32 soil-core samples from the four selected areas. The distance between each soil sampling point was approximately between 10 and 50 m. Specifically, the distance between the soil sampling points in Area A was kept as 10 m in order to evaluate the position resolution of our method. 

### 2.2. Analytical Schema of the ARM Data Obtained Using an Unmanned Helicopter

To conduct an ARM survey of the farm, a LaBr_3_:Ce detector (Japan Radiation Engineering Co., Ltd., Ibaraki, Japan) was placed under the helicopter. The total gamma-ray count rate and pulse height distribution data of 1024 channels were measured every second. Because the spectra of LaBr_3_:Ce (38.1 mm*φ* × 38.1 mm × 3 = 0.13 L) showed a good energy resolution (FWHM 1.8% at 662 keV: ^137^Cs), it was able to clearly distinguish the 605 keV energy peak of ^134^Cs from the 662 keV energy peak of ^137^Cs. Typical analytical methods for the conversion from count rate to dose rate at an altitude of 1 m above the ground were established in our previous studies [7,8,9]. First, the background gamma-ray count rate (*C_BG_*) (self-contamination of the helicopter) was subtracted from total gamma-ray count rate (*C_Gross_*); *C_BG_* was obtained at a 200-m altitude above the sea level on 1 April 2015. Then, the gamma-ray count rate (*C_Net_*) derived from the ground was defined using Equation (1) as follows:

*C_Net_* = *C_Gross_* − *C_BG_*. (1)

**Figure 3:**
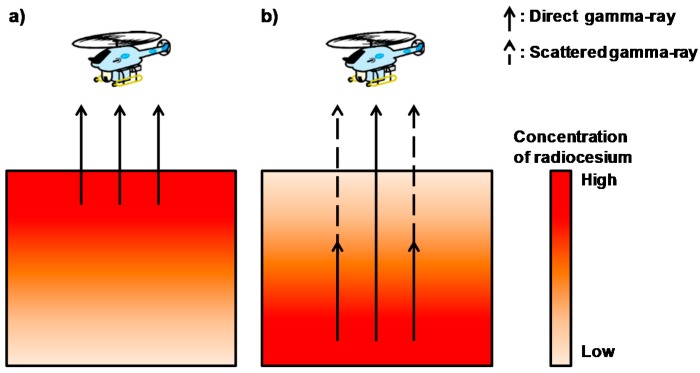
Image of gamma-rays derived from radiocesium in the soil; (**a**) normal condition and (**b**) irregular condition following inversion tillage.

**Figure 4:**
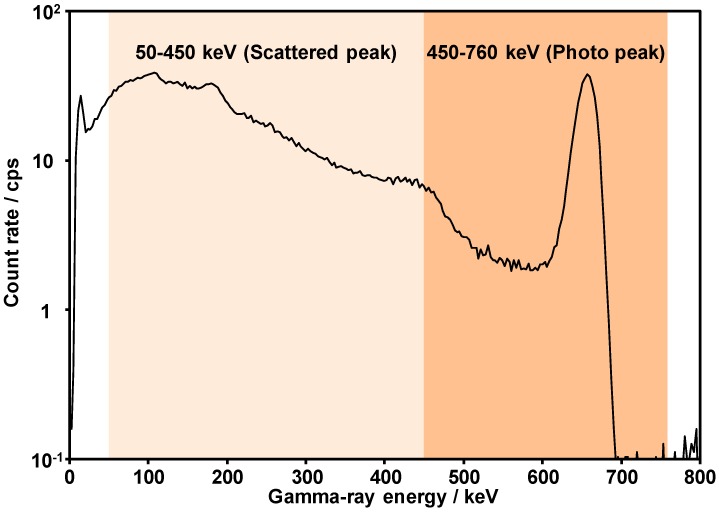
Conceptual schema for dividing the gamma-ray spectrum obtained from the LaBr_3_:Ce detector into two sections.

In general, a LaBr_3_:Ce detector detects direct gamma rays and Compton scattered gamma rays from soil particles. The deeper the radiocesium is located in the soil, the greater the ratio of scattered gamma rays to the detected direct gamma rays. An image of the gamma-ray spectra derived from radiocesium deposited on soil is shown in Figure 3. A conceptual schema of dividing the gamma-ray spectrum obtained from the LaBr_3_:Ce detector into two sections is shown in Figure 4. The gamma-ray spectra obtained from the LaBr_3_:Ce detector were divided into two sections at an energy of 450 keV in order to evaluate the radiocesium depth obtained from ARM results. The Compton count rate ratio (RPC) is defined as the ratio of the gamma-ray count rate between 50 and 450 keV (*C_50–450 keV_*) to that between 450 and 760 keV (*C_450–760 keV_*):

*RPC* = Σ*C_50–450 keV_*/Σ*C_450–760 keV_* (2)

### 2.3. Quantitative Evaluation of the Vertical Distribution of Radiocesium Using Distribution Parameters

Soil samples were collected using a liner sampler (Eijkelkamp, Giesbeek, The Netherlands) and a single gouge auger (Eijkelkamp). The soil cores were sliced into 2–6-cm-thick sections. Each soil sample was homogenized and placed into a plastic vial bottle (polyethylene, PerkinElmer Japan, Yokohama, Kanagawa, Japan) and then measured using a NaI(Tl) detector (2480 Wizard, PerkinElmer). A 75-mm-thick Pb shield was installed around the sample to reduce the intensity of the background signal. The activities of radiocesium in samples were calibrated with those of the standard materials. The measured radioactivities were corrected for decay with respect to the sampling date. 

Generally, the activity concentration of radiocesium decreased exponentially with mass depth from the surface soil because the gamma rays derived from radiocesium were attenuated by the upper-soil particles. However, in some cases, when inversion tillage or soil erosion due to rainfall occurred, this hypothesis did not hold true. In these cases, the vertical distributions of radiocesium were quantitatively expressed as a distribution parameter (*β_eff_*) using the equations presented in [18] and as follows:

(3) $$D=\int_{0}^{\infty }A_{m,0,eff}\exp\left(-\zeta /\beta_{eff}\right)I_{\gamma }C\left(\zeta \right)d\zeta ,$$

(4) $$\zeta =\int_{0}^{z}\rho \left(z^{\prime }\right)dz^{\prime },$$

*A_inv._* = *β_eff_* × *A_m,_*_0*, eff*_, (5)

where *A_m,_*_0, *eff*_ is the effective activity concentration (wet weight) at the ground surface of the soil (Bq kg^−1^); *ζ* is the relaxation mass depth (g cm^−2^); *β_eff_* is the effective relaxation mass depth (g cm^−2^), at which the concentration of radiocesium reduces to 1/e of the concentration at the ground surface; I_γ_ is the gamma-ray branching ratio; *C(ζ)* is the conversion factor proposed by Saito [20]; *D* is the air kerma rate at 1 m above the ground (*μ*Sv h^−1^), calculated using Saito’s conversion factor; *z’* is the actual depth from the ground surface (cm); *ρ* (g cm^−3^) is the soil density; and *A_inv._* is the measured deposition (Bq m^−2^). The soil density *ρ* was determined based on the mass (wet weight) and the volume of each layer of the soil samples [18].

### 2.4. Calculation of β_eff_ Based on the ARM Results

**Figure 5:**
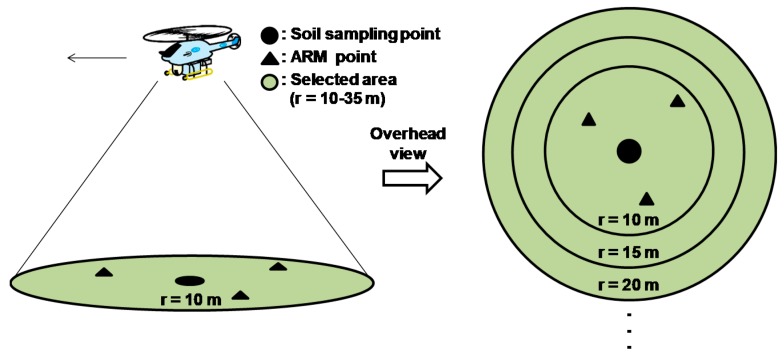
Conceptual schema for ARM data selection based on the position of soil sampling points; black dots indicate soil sampling points, black triangles indicate ARM points, and the green circle indicates the selected area based on the position of soil sampling points.

For evaluation of the *β_eff_* based on ARM results, RPC which was obtained by some conditions and compared to observed *β_eff_*. ARM was performed for the entire study site to evaluate the deposition pattern of radiocesium on the ground. Therefore, the ARM results not only reflect the specific measurement locations but also the surrounding environments, e.g., forests. It is necessary to know the valid range of gamma rays derived from radiocesium on the ground in order to detect the optimum range of the LaBr_3_:Ce detector placed under the helicopter. We defined a hypothetical circle based on the position of each soil sampling point. The radii of the circles were determined to be at distances of 10, 15, 20, 25, 30, and 35 m from each soil sampling point. RPCs were selected only from the different range circles. A conceptual schema for selecting ARM data is shown in Figure 5. These comparison schemas were applied to flight data of 20 m and 30 m agl. The parameter which can convert from RPC to *β_eff_* was determined with the most suitable coefficient correlation. The contour map of *β_eff_* based on RPC were calculated and interpolated in 28 m mesh via a Kriging method. 

## 3. Results

### 3.1. Distribution of the RPC Obtained via ARM

**Figure 6:**
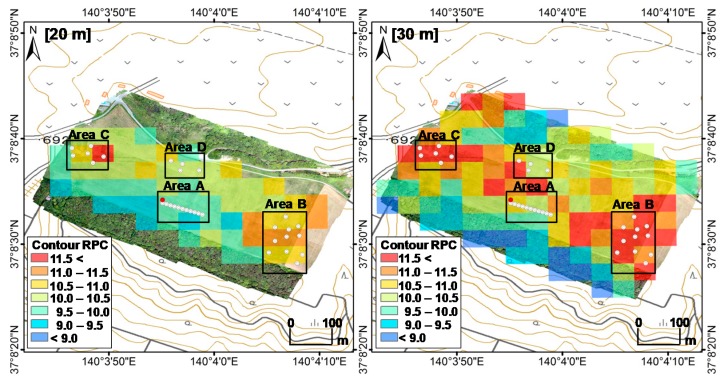
Maps of the averaged RPCs (50 m × 50 m mesh) obtained via ARM at 20 and 30 m agl.

The distribution of the RPC values obtained via ARM at 20 and 30 m agl. is shown in Figure 6. The RPC values are averages calculated within the selected area (50 m × 50 m). The RPC at 20 m agl. was lower than that at 30 m agl. owing to the scattering of gamma rays in the air. The RPC in Area A was lower than that in other areas because inversion tillage had not been performed in this area. In contrast, the RPC in Area B was uniformly high in the farm as a result of inversion tillage. The highest RPC value was observed in Area C owing to non-uniform inversion tillage. The RPC in Area D was relatively low in the parts where inversion tillage had been performed. 

### 3.2. Unnatural Vertical Distribution of Radiocesium in Soil Resulting from Inversion Tillage

**Figure 7:**
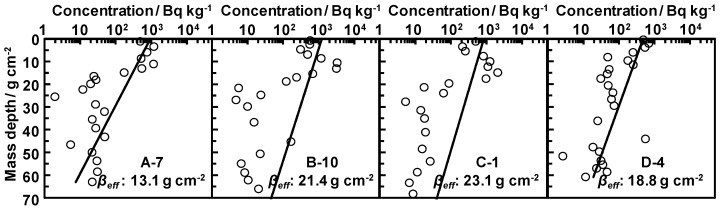
Vertical distributions of the activity concentrations of radiocesium with mass depth (wet weight) and approximate curves.

A large amount of radiocesium generally exists in surface soil [10,11,12,13,14,15,16,17,18,19]. However, unnatural vertical distributions of radiocesium were observed in the farm. Typical cases are shown in Figure 7. The experimental results suggest that the depth at which the maximum activity concentration of radiocesium was found was between 5 and 30 cm and that *β_eff_* was between 5.04 and 88.0 g cm^−2^. In general, *β* was between 0.1 and 3.0 g cm^−2^ during the first five years post the FDNPP accident [21]. The *β* value was calculated using an exponential function under normal soil conditions [18]. Therefore, the vertical distributions of radiocesium in the study site were highly affected by inversion tillage in the farm. When focusing on the features of each soil sampling area, it was found that the *β_eff_* in Area A was higher than that in areas where inversion tillage was not performed. Field observations also indicated non-uniform disturbance of the soil profile by wild animals. The *β_eff_* in Areas B, C, and D were higher than that in Area A, which was affected by inversion tillage. All the vertical distributions of radiocesium are shown in the Appendix A (*n* = 32). 

### 3.3. Relationship between RPC and β_eff_

**Figure 8:**
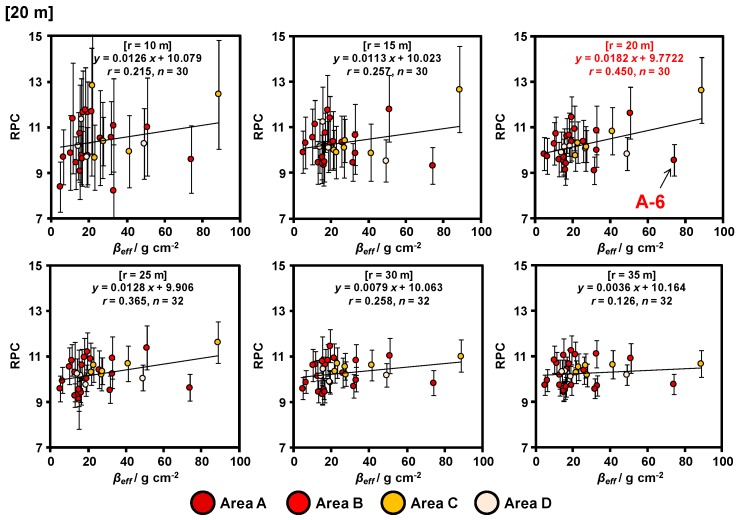
Relationship between RPC at 20 m agl. and *β_eff_* with different selected ranges based on soil sampling points.

**Figure 9:**
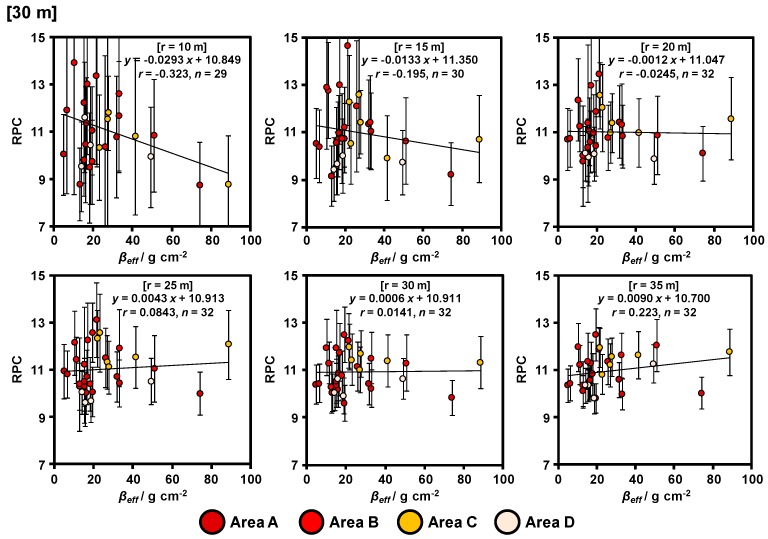
Relationship between RPC at 30 m agl. and *β_eff_* with different selected ranges based on soil sampling points.

The relationships between the RPCs obtained via ARM at 20 and 30 m agl. and the *β_eff_* calculated via actual measurements of the vertical distributions of radiocesium in the soils are shown in Figure 8 and Figure 9, respectively. The error bar for RPC represents the count rate error obtained via ARM. The count rate error was calculated focusing on the square root of gamma-ray count. The RPCs calculated from the data obtained at 30 m agl. were higher than those obtained at 20 m agl. within the same radii circles. It appears that an increase in the density of scattered gamma rays was caused by the effect of air as the flight altitude increased (Figure 6). From these results, the equations for calculation of *β_eff_* based on RPC were determined. These equations contain the uncertainty, for example, locally high data (A-6). We focused on the valid range of gamma ray and horizontal spread status of radiocesium in the Discussion section for creation more suitable *β_eff_* horizontal distribution maps in the entire study site.

## 4. Discussion

### 4.1. Estimation of the Valid Range of Gamma Ray

The RPC (11 ± 2%) calculated using the 20 m agl. ARM data in the circle (r = 20 m) was in good agreement with the *β_eff_* value. When the radii of the circle were narrow (r = 10 and 15 m), only few ARM data in the circle were available, and they reflected only the ground directly below. In contrast, when the radii of the circle were wide (r = 25, 30, and 35 m), a greater number of ARM data in the circle was available and the gamma-ray count error decreased. However, the gamma-ray spectra obtained were affected by both the ground directly below the helicopter and by the surrounding environment. Thus, we focused on ARM data in the circle (r = 20 m) for estimating the *β_eff_* value.

The number of ARM data obtained at 30 m agl. were fewer than those obtained at 20 m agl. in the same radii circle because the interval of the flight path at 30 m agl. was larger than that at 20 m agl. Therefore, the gamma-ray count error at 30 m agl. was larger than that at 20 m agl. in the same radii circle. The RPC calculated using the 30 m agl. ARM data in the circle (r = 35 m) was slightly in good agreement with the *β_eff_* value. Both the slope of the curves and the correlation coefficients increased with the radii of the circles. These results suggest that the valid range of gamma rays was different in response to the flight altitude.

### 4.2. Consideration of the Horizontal Spread of Radiocesium in the Soil

**Figure 10:**
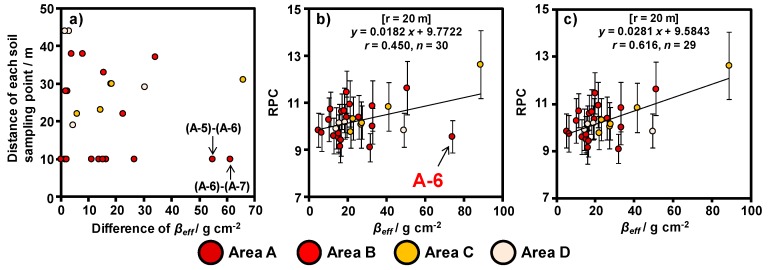
Conceptual schema showing the process for removing irregular data from the 20 m agl. dataset. (**a**) Relationship between the distance of each soil sampling points and the difference in *β_eff_*; (**b**) relationship between RPC and *β_eff_* before the removal of the irregular data; and (**c**) relationship between RPC and *β_eff_* after the removal of the irregular data.

In this section, we evaluate the horizontal spread of radiocesium in the soil based on the relationship between RPC and vertical distribution. An identification schema of irregular data (A-6) based on the distance of each soil sampling point is shown in Figure 10. In Figure 10a, the distance of each soil sampling point was calculated using data obtained from a handheld GPS device, and the difference in the *β_eff_* values was calculated by subtracting large *β_eff_* from small *β_eff_*. In particular, the *β_eff_* in A-6 was locally high when compared with adjacent data. On the other hand, the RPC was nearly equivalent to the adjacent data; *β_eff_* was 19.5 g cm^−2^ and the RPC was 10.4 in A-5, and *β_eff_* was 13.1 g cm^−2^ and the RPC was 9.59 in A-7. These results indicate that the RPC was affected both by the ground directly beneath the helicopter as well as by the surrounding environment. In addition, we attempted to remove the locally high *β_eff_* in A-6 to confirm our hypothesis. The relationship between *β_eff_* and RPC after the removal of irregular data is shown in Figure 10c. Figure 10c shows a good correlation between RPC and *β_eff_*. Therefore, our method is suitable for evaluating the vertical distribution of radiocesium in soil over wide areas, with a minimum distance of >20 m between each soil sampling point. In such a case where a locally high *β_eff_* was observed owing to non-uniform inversion tillage conducted at narrow intervals of each soil sampling point (<20 m), we need to pay attention regarding the validity of the developed method. 

### 4.3. Horizonal Distribution of β_eff_ Based on the ARM Results

The relationships between the RPCs obtained in 20 m radii circle from soil sampling point and the *β_eff_* was most suitable coefficient correlation (Figure 8). We created a *β_eff_* horizonal distribution map based on the following equation obtained via ARM (Figure 10c) because the most suitable relationship with RPC and *β_eff_* was obtained by removing the locally high data in Section 4.2:

*β_eff_* = (*RPC* − 9.5843)/0.0281. (6)

**Figure 11:**
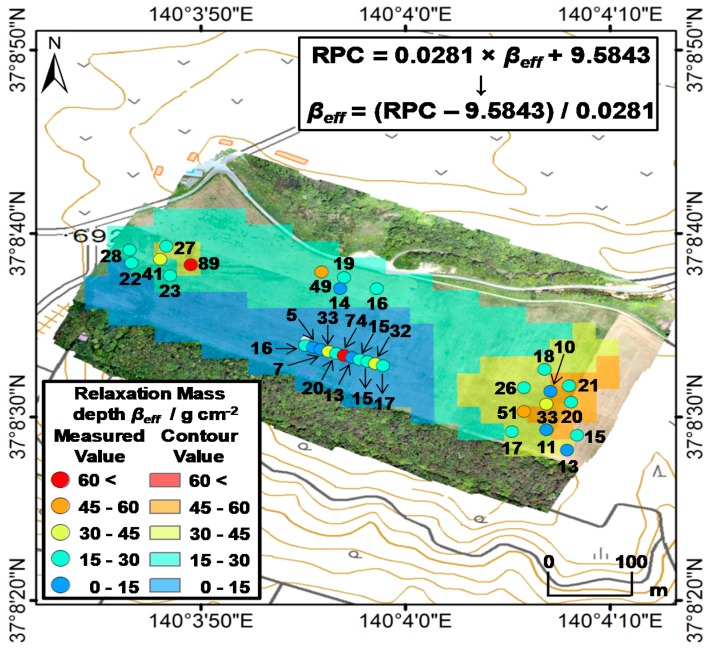
Distribution of *β_eff_* based on the RPC obtained for the farm.

The resulting map based on the equation is shown in Figure 11. The RPCs suggest that the *β_eff_* in the southern area, including Area A in the farm, is lower than those in other areas. Therefore, these results reflect the actual farm conditions, except for the locally high *β_eff_* in A-6. It is necessary to identify high-priority decontamination areas. In Area B, *β_eff_* was higher than that in other areas as a result of inversion tillage. In addition, the existence of an unusually high-*β_eff_* area was observed in Area C. It was nearly equivalent to the actual measurements of the vertical distribution of radiocesium in the soil. The *β_eff_* in Area D was relatively low at parts where inversion tillage had been performed. This study shows that our method is suitable for rapid and easy estimation of the vertical distribution of radiocesium in soil over wide areas (600 m × 180 m). 

## 5. Conclusions

In this paper, we performed ARM using an unmanned helicopter in an extended farm to develop an effective estimation method for the vertical distribution of radiocesium in soil over wide areas. The RPC was calculated by dividing the total gamma-ray count rate obtained from gamma-ray spectra into two sections based on the scattered peak (50–450 keV) and the photo peak (450–760 keV). The ARM results suggest that the RPC temporarily varied in response to inversion-tillage practices. Vertical distributions of radiocesium were investigated in situ with soil sampling to verify the hypothesis that the density of scattered gamma rays increases and that of direct gamma rays derived from radiocesium decreases with an increase in the depth of radiocesium in the soil. The vertical distribution of radiocesium was quantitatively expressed as the parameter of effective relaxation mass depth, *β_eff_*. The investigation of the actual depth of radiocesium in the soil suggests that the *β_eff_* in Areas B, C, and D was higher than that in Area A. In addition, the *β_eff_* in Area A was not constant despite the short distance between each soil sampling point because of soil disturbance caused by wild animals. In conclusion, the RPCs obtained via ARM were correlated with the *β_eff_* obtained in situ from the vertical distributions of radiocesium in the soil after removing the locally high *β_eff_* value (A-6). This study shows that the method developed herein is suitable for evaluating the vertical distribution of radiocesium in soils over wide areas when the minimum distance between each soil sampling point is >20 m. Therefore, our method provides a novel approach for effective monitoring of radiocesium depth in soils over wide areas without necessitating sampling and measurement of the activity of the soil.

## Figures and Tables

**Figure A1:**
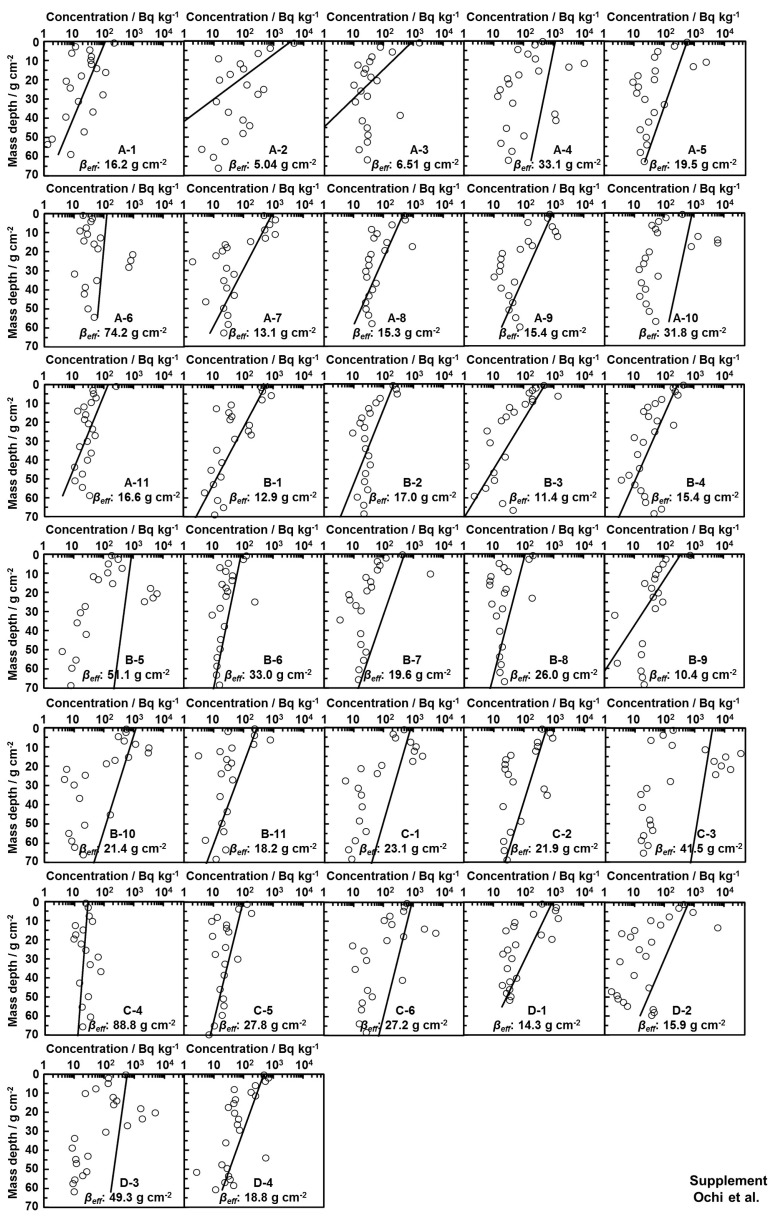
All vertical distributions of radiocesium are shown in the appendix (*n* = 32).
